# Slow proliferation as a biological feature of colorectal cancer metastasis

**DOI:** 10.1038/sj.bjc.6605229

**Published:** 2009-08-04

**Authors:** A Anjomshoaa, S Nasri, B Humar, J L McCall, A Chatterjee, H-S Yoon, L McNoe, M A Black, A E Reeve

**Affiliations:** 1 Cancer Genetics Laboratory, Department of Biochemistry, University of Otago, Dunedin, New Zealand; 2 Department of Surgery, University of Auckland, Auckland, New Zealand; 3 Department of Pathology, University of Otago, Otago, New Zealand; 4 Otago Genomics Facility, Department of Biochemistry, University of Otago, Otago, New Zealand

**Keywords:** colorectal cancer, proliferation, liver metastasis

## Abstract

**Background:**

We have recently reported an inverse relationship between colon cancer progression and tumour proliferative activity. Here, we extend our findings by evaluating the proliferative activity of liver metastatic lesions and primary colorectal cancers (CRC) that differ in their metastatic potential.

**Methods:**

Using an earlier established multi-gene proliferation signature (GPS), proliferative levels were analysed in 73 primary CRCs and 27 liver metastases.

**Results:**

Compared with primary CRCs, we observed a significantly lower expression of the GPS in liver metastases and confirmed their lower proliferative levels by quantitative RT–PCR and Ki-67 immunostaining. No difference could be detected in apoptotic indices as assessed by M30 immunostaining, indicating that the net growth rate is lower in metastases relative to primary tumours. Notably, relapsed primaries or those with established metastases had significantly lower proliferative activity than CRCs that were non-metastatic and did not relapse.

**Conclusion:**

Our results suggest that slow proliferation is a biological characteristic of both liver metastases and those primary tumours with the ability to metastasise. The delineation of the mechanisms underlying the inverse association between proliferation and CRC aggressiveness may be important for the development of new therapeutic strategies.

Metastasis is the major cause of cancer death. The underlying process consists of a series of interrelated biological steps that eventually lead to the formation of metastatic lesions. This highly selective process favours the survival and proliferation of a sub-population of cancer cells with the ability to complete all steps. In addition to these intrinsic features of metastatic cells, their interaction with the host organ is another potential determinant of successful metastasis formation (Steeg, 2006). However, our knowledge of the mechanisms driving the spread of primary tumours is limited.

Colorectal cancer (CRC) is one of the most common causes of cancer-related mortality in the Western world and metastasis to the liver is responsible for a large proportion of the deaths from CRC. Approximately 50% of CRC patients develop liver metastases during the course of their disease (Bird *et al*, 2006). Although surgery and adjuvant therapy can effectively control CRCs at the primary site, 80–90% of metastatic lesions are not resectable (Adam and Vinet, 2004). Even with access to modern chemotherapy drugs, non-operable patients have a median survival rate of less than 2 years (Wolpin and Mayer, 2008). A deeper understanding of the biology of metastasis may improve the ability to identify patients at high risk of developing metastases and lead to the discovery of better treatments for established metastatic disease.

A fundamental feature of cancer cells is their altered proliferative rate, which is often used as a basis for current chemotherapies. We, however, have recently reported an inverse relationship between the proliferative activity of primary colon cancers and their malignant potential by utilising a multi-gene proliferation signature (GPS) (Anjomshoaa *et al*, 2008). The GPS provides an objective and robust assay for the assessment of proliferation, as its expression is increased relative to quiescent cells in both (i) proliferating CRC cells *in vitro* and (ii) the proliferative region of colonic crypts *in vivo*. The GPS was tested on breast cancer, where it correctly identified the well-established association between increased proliferation and worse outcome. However, when the GPS was used to analyse biopsy material from two independent cohorts of colon cancer patients, lower cellular proliferation was found to be associated with advanced disease stages and a shorter disease-free survival. Of note, these associations were independent of adjuvant chemotherapy received by some patients.

Here, we present evidence corroborating the association between more aggressive biological behaviour and low proliferation in CRC by analysing CRC liver metastases and a series of primary tumours differing in metastatic potential. We found that both metastasising primaries and liver metastases are characterised by a reduced proliferative activity relative to non-metastatic CRCs.

## Materials and methods

### Patients and tumour samples

Tumour specimens were obtained from 73 patients undergoing surgery for primary CRC at Dunedin Public Hospital and 27 patients undergoing resection of CRC liver metastases at Auckland City Hospital, New Zealand. Fresh frozen specimens were taken from each tumour and stored at −80°C. Formalin-fixed paraffin-embedded tissue blocks were also available. Ethical Committee approval was obtained and all participants gave signed informed consent.

The primary tumours and liver metastases were not paired. Of the 27 patients operated on for liver metastases, 10 had synchronous and 17 had metachronous disease. Of the 73 primary tumours, 40 were situated in the colon and 33 were rectosigmoid or rectal. Ten had stage I, 27 stage II, 28 stage III and 8 stage IV disease. Patients were followed up for a minimum of 5 years. Of the 65 patients with primary CRC who underwent potentially curative resection (stage I–III patients), 24 developed disease relapse, 21 of which included systemic spread. Three isolated local relapses were not included in the analyses.

### Array preparation and gene expression analysis

Gene expression profiling of all tumours was performed using arrays spotted with an MWG 30K Oligo Set (MWG Biotech, Winston-Salem, NC, USA) as described previously (Anjomshoaa *et al*, 2008). Briefly, Cy dyes were incorporated into cDNAs synthesised from 10 *μ*g tumour sample or reference RNA using the indirect amino-allyl cDNA labelling method. A pooled RNA sample derived from a mixture of 12 cell lines was used as a reference for all hybridisations. The mixture of dye-labelled cDNAs was then purified and co-hybridised to a microarray. After scanning with a GenePix 4000B microarray scanner (Molecular devices, Sunnyvale, CA, USA), the foreground intensities from each channel were log_2_-transformed and normalised using the SNOMAD software (Colantuoni *et al*, 2002). Normalised values were collated and filtered using BRB-Array Tools version 3.6.0-*β*_3 (http://linus.nci.nih.gov/BRB-ArrayTools.html). Genes of low signal intensity, or for which more than 20% of measurements across tumour samples were missing, were excluded from further analysis.

### Quantitative real-time–PCR

The expression of seven randomly selected genes from the GPS (*MAD2L1*, *POLE2*, *CDC2*, *MCM6*, *MCM3*, *PBK* and *GMNN*) and two well-known proliferative genes (*PCNA* and *Ki-67*) was validated by quantitative real-time–PCR (qRT–PCR) on an ABI Prism 7900HT system, Applied Biosystems, Foster City, NC, USA. Relative fold changes were calculated using the 2^−ΔΔ*C*T^ method (Livak and Schmittgen, 2001). Topoisomerase 3A (*TOP3A*) was used as the internal control because it had the least expression variation across samples compared with other potential housekeeping genes (i.e. *ACTB*, *DSP*, *BAZ2A*). Reference RNA (the same reference as for microarrays) was used as a calibrator to enable comparison between different experiments.

### Immunohistochemistry

Immunohistochemical assessment of proliferation and apoptosis was performed in all tumours. Antigens were retrieved on 4 *μ*m sections in boiling citrate buffer (pH 6). Primary antibodies (MIB-1/Ki-67 and M30 Cytodeath; dilution 1 : 50; Dako Glostrup, Denmark) were detected using the EnVision system (Dako) and the DAB substrate kit (Vector Laboratories, Burlingame, CA, USA). For each tumour sample, cells within five high-power fields were counted by two observers in a blinded manner. The proliferative and apoptotic indices were presented as percentage of positively stained cells.

### Statistical analysis

All microarray analyses were performed using BRB-Array Tools software. Testing for significant differences in the GPS expression or immunohistochemical indices between groups of tumours was carried out with SPSS 15.0.0 (SPSS Inc., Chicago, IL, USA). A multiple comparisons correction (Bonferroni) was applied to the *P*-values derived from testing for differences in microarray-based gene expression. Additional details relating to the statistical methods and their specified parameters are described in the corresponding results sections.

## Results

### Lower GPS expression in liver metastases compared with primary CRCs

To investigate whether liver metastases and primary CRCs differ in their proliferative activity, the expression of genes constituting our GPS was compared between the two tumour groups. Expression values for the GPS genes were obtained from the array-generated expression profiles. Expression data were available for 33 out of the 36 GPS genes. *CDCA5*, *KIF4A* and *GINS2* were filtered out due to the poor quality of spots. We first examined whether GPS expression differs between tumours of rectal and colonic origin. Because the GPS levels were similar in the rectal and colonic groups (adjusted *P*>0.4, two-tailed *t*-test based on 1000 permutations), rectal and colon tumours were considered as one group in all subsequent analyses. Intriguingly, 30 out of the 33 GPS genes had significantly lower expression in liver metastases compared with primary CRCs (adjusted *P*<0.01; two-tailed *t*-test) (Supplementary Table 1). Only three genes (*CDC20*, *NOLA2* and *SSX2IP*) had similar expression levels. These results suggest that liver metastases are less proliferative than primary CRCs.

### Lower expression by qRT–PCR of GPS genes in liver metastases compared with primary CRCs

**Figure 1:**
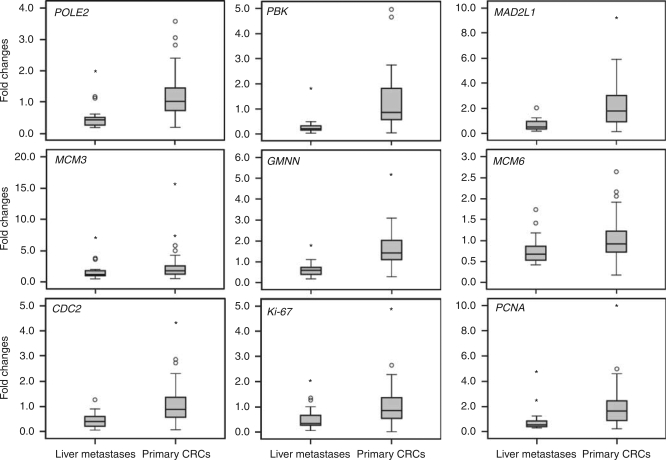
Quantitative RT–PCR confirmed the lower expression of nine cell-cycle-related genes in liver metastases compared with primary CRCs. The *Y* axis represents the fold changes calculated as the ratio of tumour to reference gene expression, normalised to a control gene. The box range (interquarter range) contains the middle 50% of the data. The median value is shown as a horizontal line across the box. The extreme values (within 1.5 times the interquartile range from the upper or lower hinges) are the ends of the whiskers. Points at a greater distance than 1.5 and 3 times of the interquartile range are outliers and presented as circles and asterisks, respectively. All differences were significant at the *α*-level of 0.005 using the Mann–Whitney *U*-test. Analysis was performed using SPSS software.

To validate the microarray data, seven genes from the GPS (*MAD2L1*, *POLE2*, *CDC2*, *MCM6*, *MCM3*, *PBK* and *GMNN*) and two established proliferation-related genes (*PCNA* and *Ki-67*) were assessed by qRT–PCR. As shown in Figure 1, all examined genes displayed significantly lower expression in liver metastases relative to primary CRCs (*P*<0.005; Mann–Whitney *U*-test). Strong (correlation coefficients 0.74–0.86) and highly significant (*P*<0.001; Spearman's correlation test) correlation was observed between the microarray- and qRT–PCR-generated expression data.

### Lower Ki-67 labelling index in liver metastases compared with primary CRCs

**Figure 2:**
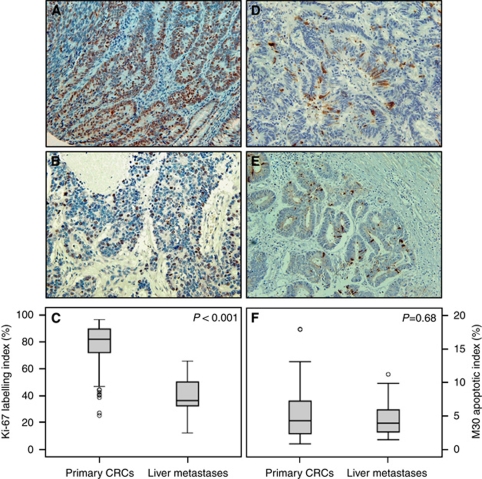
Nuclear staining of Ki-67 protein in a primary CRC (**A**) and a liver metastasis (**B**). Cytoplasmic staining of M30 apoptotic protein in a primary CRC (**D**) and a liver metastasis (**E**). The Ki-67 LI was significantly lower in liver metastases compared with primary CRCs (**C**). No significant difference was observed in M30 AI between liver metastases and primary CRCs (**F**). *P*-values were calculated using the Mann–Whitney *U*-test.

To determine whether liver metastases and primary CRCs differ in Ki-67 nuclear expression, the Ki-67 labelling index (LI) was compared between the two groups (Figure 2A and B). Median (range) Ki-67 LI was 81.8% (25.1–96.4%) in primary CRCs, and 36.2% (12.1–65.5%) in liver metastases (*P*<0.001; Mann–Whitney *U*-test; Figure 2C).

### Similar M30 apoptotic index in liver metastases and primary CRCs

To determine whether liver metastases and primary CRCs differ in apoptotic rates, the M30 apoptotic index (AI) was assessed in the two groups (Figure 2D and E). Median (range) M30 AI was 4.28% (0.83–17.95%) in primary CRCs and 3.91% (1.45–11.2%) in liver metastases. No significant difference in the M30 AI was detected between primary CRCs and liver metastases (*P*=0.68; Mann–Whitney *U*-test; Figure 2F). The observation of lower proliferative index of liver metastases relative to primaries, together with a similar AI, indicates that the net growth rate is lower in liver metastases than in primary CRCs.

### Slow proliferation is a biological feature of CRC metastasis

**Figure 3:**
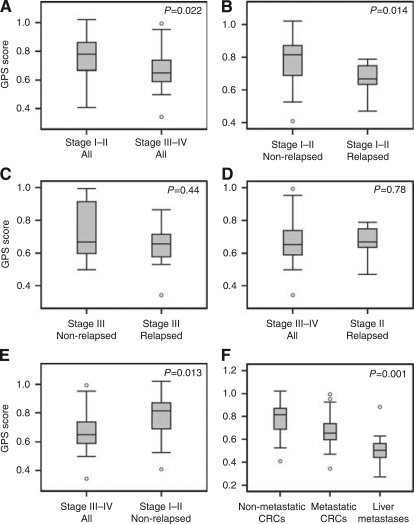
Box plots summarising the distribution and variability of GPS scores in defined classes of CRCs. GPS expressions for 33 genes (log_2_ target/reference) were first averaged for each sample, and then transformed into fold changes as GPS scores. The exact levels of significance were calculated using the Exact method implemented in Mann–Whitney *U*-test (for comparison of two groups) and Kruskal–Wallis test (for comparison of more than two groups). Resulting *P*-values are indicated in the right upper part of each panel. Numbers in the upper left of plots refer to the corresponding paragraphs in the manuscript. The interpretation of box plots has been described in the legend of Figure 1.

Because metastatic lesions are the result of a process that is initiated at the primary tumour site, they should share biological features with primary tumours that have a high metastatic potential. We therefore hypothesised that primary CRCs with a high metastatic potential have slower proliferative rates than CRCs with a low metastatic potential. To test this hypothesis, GPS genes were first averaged to assign each tumour a mean GPS score. GPS scores were then compared between groups of tumours classified according to the tumour stage or relapse status. *P*-values were calculated using the Exact test option in SPSS, which provides more accurate estimates of significance for non-parametric tests when dealing with small sample sizes. The Mann–Whitney *U*-test was used for comparison of two groups and Kruskal–Wallis test for comparison of more than two groups. The results are summarised below:

- To determine whether tumour proliferation is related to the presence of metastasis at the time of surgery, GPS scores were compared between stage I–II and stage III–IV primary tumours. Stage I and II CRCs were grouped as they were confined to the intestinal wall and no difference in proliferation was detected between non-relapsed stage II and I tumours (*P*=0.77). Stage III and IV CRCs were grouped because these two stages of disease did not differ in proliferation (*P*=0.92). Stage III–IV tumours had significantly lower GPS scores than stage I–II tumours (*P*=0.022; Figure 3A). This analysis suggests that CRCs with established metastasis to the lymph nodes or distant organs have lower proliferative activity than CRCs that were non-metastatic at the time of surgery.
- To determine whether slow proliferation in stage I–II tumours was associated with disease relapse, GPS scores were compared between the tumours of patients who remained relapse-free and those who relapsed. All stage I tumours remained relapse-free, while 7 out of 27 stage II tumours experienced relapse during a 5-year follow-up. The GPS scores were found to be significantly lower in the relapsed compared with the non-relapsed group (*P*=0.014; Figure 3B). When analysis was limited to stage II tumours, the association between tumour proliferation and relapse remained significant (*P*=0.035).
- To determine whether proliferation is associated with the likelihood of relapse in tumours with lymph node metastasis at the time of surgery, only stage III CRCs were analysed. No difference in the GPS scores was detected between 11 relapse-free tumours and 14 tumours that relapsed after surgery (*P*=0.44; Figure 3C). Three tumours had local relapse and were not included in the analysis. Stage IV tumours were not analysed as all had residual disease from the time of surgery. These results suggest that low proliferation is not associated with the likelihood of relapse but rather with the ability to disseminate beyond the primary site.
- To determine whether slow proliferation is a common feature of primary tumours with metastasis at the time of surgery and metastasis during follow-up, the GPS scores of stage III–IV and stage II CRCs that relapsed during follow-up was compared. Interestingly, no difference in the GPS scores was detected (*P*=0.78; Figure 3D), indicating that slow proliferation is part of a metastatic signature common to both. These results confirm that slow proliferation is associated with the ability to metastasise as assessed under (C).
- To further confirm the association between slow proliferation and metastatic potential in primary tumours, relapse-free stage I–II tumours and stage III–IV tumours were analysed. As expected, GPS scores were significantly lower in stage III–IV tumours compared with the other group (*P*=0.013; Figure 3E).
- To determine whether CRC proliferative activity inversely correlates with increasing metastatic potential, we compared GPS scores between three groups of tumours. Group 1 (non-metastatic CRCs) included stage I–II tumours from patients who remained relapse-free. Group 2 (metastatic CRCs) included stage III–IV and stage II tumours from patients who developed relapse. Group 3 included the liver metastases. GPS scores were significantly different between the three groups (*P*=0.001; Figure 3F). Proliferative activity increased from liver metastases to metastatic primaries and was highest in non-metastatic primaries, confirming that slow proliferation is related to the ability of CRCs to metastasise.

### Gene set comparison analysis confirms the significant differences in the GPS between tumour groups differing in their metastatic potential

To confirm the significant differences in proliferative activity of the tumour groups defined above (A–F), we used gene set comparison analysis as an alternative approach. When comparing different tumour groups, the GPS was considered to have a higher than expected number of differentially expressed genes if the Kolmogorov–Smirnov (KS) re-sampling *P*-value was less than 0.005 (default value). The distribution of KS statistics was obtained following 100 000 random re-sampling events. Gene set comparison analysis confirmed that the GPS contained a higher than random proportion of DE genes among groups as was observed with the non-parametric tests (data not shown).

Altogether, the comparison of GPS levels in the various CRC groups strongly suggests that slow proliferation is a biological feature of CRCs that have the potential to metastasise.

## Discussion

Dysregulated cellular proliferation is a defining characteristic of cancer. It is therefore not surprising that cell proliferation has received considerable attention in the field of cancer biology and has been extensively studied as a means to predict the behaviour of tumours. The general perception says that a selective growth advantage is fundamental to the development and progression of cancers. Many studies support this concept by demonstrating that more aggressive tumours have a higher proliferative capacity (Rosenwald *et al*, 2003; Krasnoselsky *et al*, 2004; Dai *et al*, 2005). However, we have recently reported that the opposite is true in colon cancer (Anjomshoaa *et al*, 2008).

In the present study, the significantly lower proliferative activity of liver metastases relative to primary CRCs provides additional evidence for a correlation between slow proliferation and high aggressiveness of CRC. Our conclusion is based on the differential expression of multiple proliferation-associated genes (the GPS) between liver metastases and primary CRCs, which was confirmed by single-gene quantitative RT–PCR assays. Furthermore, Ki-67 immunostaining confirmed the low levels of proliferation in CRC metastases and demonstrated that the difference in the proliferative activity between liver metastases and primary CRCs was large enough to be detected by a subjective single-protein assay. Finally, the combination of a lower proliferative activity with a similar AI in metastases relative to primaries is consistent with the clinical observation that liver metastases are sometimes slow growing. Other groups using immunohistochemical methods have come to similar conclusions (Thomas *et al*, 1998; Agui *et al*, 2002; Kitabatake *et al*, 2002; Seong *et al*, 2004).

An important observation in our study is that low relative proliferation appears to be a biological feature of CRCs with a high metastatic potential. This viewpoint is strongly supported by the GPS-based analysis of primary CRCs, where groups of highly malignant tumours (high stage and relapse) displayed low proliferative activity relative to less malignant tumours (low stage and low risk of relapse). All observed associations between metastatic capacity of primary CRCs and their proliferative activity also hold up in the cohort of 108 tumours analysed in our earlier study (Anjomshoaa *et al*, 2008). The low proliferation of metastatic CRCs was intermediate between that of non-metastatic CRCs and liver metastases, further strengthening this view. This is consistent with the expression profiling studies that have demonstrated a substantial signature overlap between metastases and their original primaries, but a lack thereof between metastatic and non-metastatic primaries (Koehler *et al*, 2004; D’Arrigo *et al*, 2005). We therefore propose that reduced proliferative activity is part of a biological signature that renders CRCs aggressive.

There are some strengths and weaknesses in this study. Strengths include the multi-gene method of assessing proliferation, which has been validated earlier (Anjomshoaa *et al*, 2008) and was further validated in this study by RT–PCR and an established immunohistochemical method for the measurement of proliferation. Another strength is the long and complete follow-up in our cohort of patients with primary CRC, which minimises the risk of mis-classification when separating the groups according to relapse. A weakness of the study is that the 27 patients with liver metastases all underwent liver resection, yet the majority of patients with liver metastases from CRC are not resectable. This cohort may thus not be representative of the broader group with liver metastases. This limitation was imposed because it is difficult to obtain fresh tumour samples from patients with unresectable liver metastases. Nonetheless, it seems unlikely that the biological behaviour of resectable liver metastases will significantly differ from that of non-resectable ones with respect to the relative proliferation levels. Furthermore, the fact that the primaries and metastatic lesions were not matched limits the assessment of the relative proliferation differences. In addition, although we could not detect any difference in proliferation between tumours with metastasis to the lymph nodes and those with distant organ metastasis, this result remains speculative because of the small numbers of stage IV tumours.

Given that metastasis is a highly selective process and distant metastases arise from a small sub-population of aggressive cells within the heterogeneous primary lesion, it is conceivable that the biological features of the original cells will manifest themselves in the metastatic lesion. If the cells of origin are slow proliferating and resistant to apoptosis, the cells at the metastatic sites are likely to be as well. Consistent with this scenario are the intermediate proliferative levels of metastatic primaries (a sub-population of slow-growing cells within a heterogeneous tumour) and the low levels of variation among the GPS genes in the metastases (where a single invading cell has produced a clone at a distant site; see also Figure 1). In this context, the ability to survive in the secondary organ may dominate the need to proliferate fast. However, inhibitory growth signals and an inappropriate (non-physiological) environment at the host organ site may also contribute to the slow proliferation of liver metastases (Hara *et al*, 2000).

There are a number of plausible mechanisms that could underlie the association between low cellular proliferation and disease progression in CRC. Although it is intuitively logical that accelerated cell division allows for more genetic errors to accumulate and to promote tumour progression, slow proliferation can be a feature of cancer cells with the ability to spread. For example, the presence of dissociated, dedifferentiated tumour cells (budding cells) at the tumour–host interface correlates with an enhanced malignant potential in CRC (Prall, 2007). It is noteworthy that budding cells have undergone an epithelial–mesenchymal transition (EMT) and are growth arrested due to increased expression of the cell-cycle-dependent kinase inhibitor, *P16* and other unknown mechanisms (Jung *et al*, 2001). Furthermore, low cellular proliferation at the invasive margin has been associated with poor prognosis in CRC (Palmqvist *et al*, 1999). Therefore, CRCs with a high proportion of dedifferentiated cells or with an extensive tumour–host interface may be more slowly proliferating but aggressive.

Tumour cells at the tumour–host interface not only express EMT-associated genes, but also increase the expression of stemness-associated genes and therefore are considered to be migratory cancer stem cells (Brabletz *et al*, 2005). Cancer stem cells are relatively quiescent (Boman *et al*, 2008), raising the possibility that the metastatic potential of slow-proliferating CRCs may be due to the presence of a high proportion of cancer stem cells.

Another possible reason for the association between low cellular proliferation and poor prognosis could be related to chronic and acute hypoxic regions present in CRCs. It has been shown that hypoxic conditions may slow down the growth rate of tumours but can promote the onset of an EMT leading to invasion (Yang *et al*, 2008).

Aneuploidy, an abnormal chromosome number, may provide an additional explanation for the association of low proliferation with high metastatic potential. Aneuploidy would be expected to have dramatic consequences on basic cellular functions such as proliferation. In budding yeast, for example, aneuploidy in the form of extra chromosome gains was found to result in a proliferative disadvantage (Torres *et al*, 2007). This same phenomenon occurs when mammalian cells carry additional chromosomes. In a recent publication, trisomic mouse embryonic fibroblasts were shown to proliferate more slowly than the euploid controls (Williams *et al*, 2008). Furthermore, a high level of chromosomal instability inhibits tumour growth, probably owing to inefficient cell proliferation or apoptotic induction (Weaver *et al*, 2007; Garcia-Higuera *et al*, 2008). All these studies suggest that aneuploidy hampers cell proliferation in human cells. These findings along with the established association between aneuploidy and a bad outcome in CRC (Araujo *et al*, 2007) suggest that the aneuploid state of more aggressive CRCs is one likely (but by no means the only) cause for the low proliferative activity of metastatic CRC. Possible underlying mechanisms are currently under investigation in our laboratory.

The extremely poor survival for patients with metastatic disease highlights the need for improved treatments. This requires the elucidation of the molecular and cellular mechanisms underlying CRC metastasis. The intriguing finding of an inverse relationship between tumour proliferation and metastasis suggests that slow proliferation is an important biological feature of a metastatic signature in CRC. This suggests that cytotoxic drugs killing fast-proliferating cells may not necessarily be the best choice for the treatment of metastatic disease. Therefore, the delineation of the mechanisms that underlie this biological feature of CRC may lead to the development of novel antimetastatic treatment strategies.

## Conflict of interest

Anthony E Reeve is a director of Pacific Edge Biotechnology Ltd, Dunedin, New Zealand.
