# Supplementary material for: Slow proliferation as a biological feature of colorectal cancer metastasis
Source: Br J Cancer. 2009 Aug 4;101(5):822–8. doi: 10.1038/sj.bjc.6605229 (PMC2736847; doi:10.1038/sj.bjc.6605229)
Supplement: Supplementary Table 1 [file 6605229x1.doc]

| **Gene symbol** | **Llid** | **Gene description** | **Fold difference**  **(CRC/LM) ¶** | ***P*-value §** |
| --- | --- | --- | --- | --- |
| *MCM3* | 4172 | minichromosome maintenance deficient 3 | 1.5 | 4.45E-10 |
| *TYMS* | 7298 | thymidylate synthetase | 1.5 | 2.15E-09 |
| *MYBL2* | 4605 | v-myb myeloblastosis viral oncogene homolog -like 2 | 1.6 | 9.02E-09 |
| *MCM6* | 4175 | minichromosome maintenance deficient 6 | 1.9 | 9.08E-09 |
| *TTK* | 7272 | TTK protein kinase | 1.7 | 9.32E-09 |
| *MLF1IP* | 79682 | MLF1 interacting protein | 1.8 | 4.79E-08 |
| *ATPBD1C* | 51184 | ATP binding domain 1 family, member C | 1.5 | 1.20E-07 |
| *POLE2* | 5427 | polymerase , epsilon 2 | 1.4 | 2.06E-07 |
| *RRM2* | 6241 | ribonucleotide reductase M2 polypeptide | 1.7 | 2.43E-07 |
| *GMNN* | 51053 | geminin | 1.7 | 5.90E-07 |
| *CCNA2* | 890 | cyclin A2 | 1.6 | 8.23E-07 |
| *RFC4* | 5984 | replication factor C4 | 1.4 | 1.05E-06 |
| *PAICS* | 10606 | phosphoribosylaminoimidazole carboxylase | 1.5 | 1.47E-06 |
| *DKFZp762E1312* | 55355 | hypothetical protein DKFZp762E1312 | 1.5 | 2.94E-06 |
| *CDC2* | 983 | cell division cycle 2 | 1.8 | 3.14E-06 |
| *NEK2* | 4751 | NIMA -related kinase 2 | 1.4 | 5.40E-06 |
| *RUVBL1* | 8607 | RuvB-like 1 | 1.3 | 1.03E-05 |
| *RPL35* | 11224 | ribosomal protein L35 | 1.4 | 3.52E-05 |
| *PRRX1* | 5396 | paired related homeobox 1 | 1.4 | 6.67E-05 |
| *CDCA3* | 83461 | cell division cycle associated 3 | 1.4 | 7.23E-05 |
| *IL17RB* | 55540 | interleukin 17 receptor B | 1.5 | 9.12E-05 |
| *MAD2L1* | 4085 | MAD2 mitotic arrest deficient-like 1 | 1.5 | 1.35E-04 |
| *PBK* | 55872 | PDZ binding kinase | 1.5 | 3.00E-04 |
| *MRPL3* | 11222 | mitochondrial ribosomal protein L3 | 1.3 | 7.80E-04 |
| *NME1* | 4830 | non-metastatic cells 1 | 1.5 | 8.53E-04 |
| *ALG8* | 79053 | asparagine-linked glycosylation 8 homolog | 1.3 | 1.10E-03 |
| *DKC1* | 1736 | dyskeratosis congenita 1, dyskerin | 1.3 | 2.20E-03 |
| *ATIC* | 471 | 5-aminoimidazole-4-carboxamide ribonucleotide  formyltransferase/IMP cyclohydrolase | 1.4 | 4.20E-03 |
| *PLK1* | 5347 | polo-like kinase 1 | 1.3 | 4.80E-03 |
| *ITGB3BP* | 23421 | integrin beta 3 binding protein | 1.3 | 8.00E-03 |
| *SSX2IP* | 117178 | synovial sarcoma, X breakpoint 2 interacting protein | 1.2 | 0.02 |
| *NOLA2* | 55651 | nucleolar protein family A, member 2 | 1.1 | 0.1 |
| *CDC20* | 991 | CDC20 cell division cycle 20 homolog | 1.05 | 0.46 |
| *CDCA5* | 113130 | cell division cycle associated 5 | NA | NA |
| *GINS2* | 51659 | DNA replication complex GINS protein PSF2 | NA | NA |
| *KIF4A* | 24137 | kinesin family member 4A | NA | NA |

¶ Microarray gene expression fold difference between primary CRCs and liver metastases (LM)

§ Significant difference in gene expression between primary CRCs and liver metastases was determined by

adjusted Bonferroni correction, two-tailed T-test.

NA; not applicable. Expression values were not available for three genes (*CDCA5, GINS2, KIF4A*).
